# Centering healthcare workers in digital health design: Usability and acceptability of two-way texting to improve retention in antiretroviral therapy in a public HIV clinic in Lilongwe, Malawi

**DOI:** 10.1371/journal.pdig.0000480

**Published:** 2024-04-03

**Authors:** Maryanne Mureithi, Leah Ng’aari, Beatrice Wasunna, Christine Kiruthu-Kamamia, Odala Sande, Geldert Davie Chiwaya, Jacqueline Huwa, Hannock Tweya, Krishna Jafa, Caryl Feldacker

**Affiliations:** 1 Medic, Nairobi, Kenya; 2 Lighthouse Trust, Lilongwe, Malawi; 3 International Training and Education Center for Health, Lilongwe, Malawi; 4 Department of Global Health, University of Washington, Seattle, Washington, United States of America; 5 Medic, Seattle, Washington, United States of America; 6 International Training and Education Center for Health, Department of Global Health, University of Washington, Seattle, Washington, United States of America; Iran University of Medical Sciences, IRAN (ISLAMIC REPUBLIC OF)

## Abstract

New initiates on antiretroviral therapy (ART) are at high risk of treatment discontinuation, putting their health at risk. In low- and middle-income countries, like Malawi, appropriate digital health applications (apps) must fit into local clinic, connectivity and resource constraints. We describe the human centered design (HCD) and development process of an open-source, hybrid, two-way texting (2wT) system to improve ART retention. We detail the critical role of diverse healthcare workers (HCWs) in the HCD process to inform app usability, create buy-in, and ensure appropriate optimization for the local context. We optimized 2wT usability and acceptability over three HCD phases: 1) informal feedback sessions with diverse 2wT stakeholders, 2) a small pilot, and 3) key informant interviews. Phase one included four sessions with diverse HCWs, including “expert ART clients”, clinical, technical, supervisory, and evaluation teams to inform 2wT design. In phase 2, a small pilot with 50 participating ART clients aimed to inform implementation improvement. Phase three included interviews with ten HCWs to deepen understanding of 2wT acceptability and usability, documenting strengths and weaknesses to inform optimization. Multi-phase feedback sessions with HCWs helped refine 2wT language and message timing for both weekly and tailored client-specific visit reminders. The pilot led to improvements in educational materials to guide client responses and ease interaction with HCWs. In interviews, the HCWs appreciated the HCD co-creation process, suggested ways to increase access for low-literacy clients or those without consistent phone access, and felt integrating 2wT with other eHealth platforms would improve scalability. Inclusion of HCWs across phases of HCD design, adaption, and optimization increased 2wT usability and acceptability among HCWs in this setting. Engaging HCWs into 2wT co-ownership from inception appears successful in co-creation of an app that will meet HCW needs, and therefore, enhance support for 2wT clients to attend visits and remain in care.

## Author summary

During the first year on antiretroviral treatment (ART), clients are at higher risk of poor retention, putting their health at risk. To help improve client retention and, therefore, client outcomes, healthcare workers (HCWs) and healthcare systems need user-centred digital innovations that are appropriate for low- and middle- income country settings where many large-scale ART programs operate. Using a co-creation approach with HCWs from clinical, technical, supervisory, and data teams, we designed, developed, and optimized a two-way text-based (2wT) system of tailored motivation messages and visit reminders for new ART initiates (within 6 months of starting ART) at Lighthouse Trust’s public ART clinic in Lilongwe, Malawi. The 2wT application leveraged the open-source, Community Health Toolkit, and was designed based on evidence of previous 2wT success in client engagement. We describe and share results from three formative stages of app co-design: feedback sessions, a pilot, and key informant interviews with HCWs. We detail findings on the 2wT system design, perceived strengths, weaknesses, and recommendations for improvement to inform continued optimization for scale up. Overall, the co-creation approach with HCWs appears successful in designing an app that will meet HCW needs and, therefore, support early client retention in ART care.

## Introduction

In sub-Saharan Africa (SSA), retention in antiretroviral therapy (ART) is an increasing challenge that threatens both individual health and attainment of UNAIDS targets to end the AIDS epidemic by 2030 [1]. With significant healthcare system constraints and healthcare worker (HCW) shortages, ART services in SSA struggle to meet ambitious global UNAIDS 95-95-95 targets where 95% people living with HIV know their status, 95% of those diagnosed with HIV infection receive sustained ART and 95% of those on ART have their viral load suppressed. In Malawi, a recent public health survey found UNAIDS target achievement at 88-87-83 [2], demonstrating a gap in attainment of critical milestones. Evidence suggests digital health interventions, especially mobile health (mHealth) that focuses on cell phones, for direct provider to client engagement with clients on antiretroviral therapy (ART) are highly acceptable and contribute to improved compliance with ART visits [3–7], an important component of retention in care. However, not all mHealth interventions are effective for all populations at all times [8–10], and even well-designed, well-researched interventions might not be effective [11–13]. Malawi’s mobile connectivity index—a composite of infrastructure, affordability, consumer readiness, content and services—was 34.2% in 2022 versus a SSA and Europe/Central Asia index of 25.2% and 79.4% respectively, suggesting that digital health interventions in Malawi may face barriers in digital innovation success [14]. Recent guidance from the World Health Organization (WHO) on app development for global health suggests that strengthened efforts to engage diverse users in app co-design may help improve mHealth effectiveness even in low resource settings [15]. A core suggestion from the WHO guidance is to increase engagement of local HCWs and clients in app design from inception to help identify the right intervention for the specific context [16]. Adherence to those global guidelines, including emphasis on HCW user inputs, may help increase the likelihood of bringing innovations to scale [17].

Participatory human centered design (HCD) can be used to develop digital innovations that are appropriate for low- and middle-income countries (LMIC) settings where resources may be limited and ART programmatic needs are high. HCD informs digital solutions by enhancing client engagement for complex health care needs and ensuring that innovations are user-friendly for local HCWs [18–22]. HCD employs a specific mind- and skill-set that places users at the centre of the problem-solving process and values lived experiences to develop solutions that are meaningful to a given user context [23–24]. The co-creation process promotes gathering of feedback, analysis, and incorporation of diverse user inputs in system optimization to respond to user’s evolving needs, skills and behaviour during the product life cycle. Hallmarks of quality HCD-led digital innovations for HCWs are ease of use, ease of learning, and adaptability to respond to changing HCW user needs [16]. To assess those hallmarks, usability and acceptability among HCWs is critical [25]. *Acceptability* focuses on how an intervention meets users’ demand and requirements to address an identified gap (does it meet needs?) whereas *usability* includes the ease and usefulness of an intervention to fill those gap (can I use it easily and correctly?) [26]. Formative research and usability testing with diverse HCW users guides early, and repeated, feedback.

Lighthouse Trust, the largest public provider of ART in Malawi, operates five clinics across the country in Blantyre, Lilongwe, Mzuzu, and Zomba in collaboration with the Malawi Ministry of Health (MoH) and supports over 65,000 clients in care. In Lilongwe, Lighthouse Trust operates two high volume, flagship clinics: the Lighthouse Clinic and Martin Preuss Centre (MPC), with a combined 35,000 ART clients. Both clinics employ a real-time electronic medical records system (EMRS) and implement a resource-intensive client retention program, Back to Care. The program successfully traces clients who miss ART visits by ≥14 days (presumed lost to follow up [LTFU]) via phone or home visit. The Back to Care program has been successful [27–31], but gaps are growing as client volume increases. MPC clinic, alone, has over 7,800 monthly visits: approximately 10 percent of MPC clients are referred to the Back to Care program per month. High demand for the program, coupled with scarce resources, results in tracing delays and ART interruptions.

Therefore, to address gaps in adherence to ART visits for new ART initiates (within 6 months of starting ART) and reduce the Back to Care program workload, the University of Washington’s International Training and Education Center for Health (I-TECH), Lighthouse Trust, and an open-source technology partner, Medic (https://medic.org/), applied HCD to design, develop, test, and implement an innovative, appropriate, client retention system using two-way texting (2wT) between new ART clients and HCWs, aiming to increase client engagement and retention in care. 2wT builds on successful technology developed in Zimbabwe for voluntary medical male circumcision (VMMC) that found that 2wT-based follow-up between providers and clients in lieu of routinely scheduled post-operative visits was safe for clients [32]; reduced provider workload and costs without compromising care quality [33]; and enhanced client-to-clinician communication, demonstrating high usability for both VMMC clients and healthcare providers [34]. 2wT for VMMC is scaling in Zimbabwe, reaching more than 33,000 males to date [35] and is expanding for VMMC follow-up in South Africa [36,37].

Using a co-creation approach between the clinical, technical, supervisory, and evaluation teams, we adapted 2wT for ART retention, designing a hybrid 2wT system of individualized reminders and motivational messages for new ART initiates at MPC clinic in urban Lilongwe, Malawi. The aim of the Lighthouse 2wT system is to improve early ART client retention, defined as on-time attendance at ART dispensing visits in their first year on ART. 2wT was based on the open-source Community Health Toolkit (CHT) and used widely-available SMS technology for the low resource settings. As HCWs perspectives are critical for long-term app usability, buy-in and optimization, we sought to engage HCWs in 2wT app co-creation from conceptualization.

To complement previous research that found high usability and acceptability among ART clients enrolled in 2wT at MPC [38], the objective of this paper is to detail the crucial role that HCWs, themselves, play in the HCD co-creation process in developing an acceptable and useful app for client retention in a routine ART setting. We present findings from iterative formative research across three activity phases: 1) multiple HCD feedback sessions with 2wT stakeholders to inform the 2wT prototype; 2) a small pilot with 2wT clients; and, 3) key informant interviews (KIIs) focused on 2wT usability and acceptability with ten HCWs. We provide lessons learned and key successes that may inform co-design, optimization and scale-up of 2wT or other digital health innovations to strengthen ART retention in routine, high-volume, public settings in Malawi or in SSA.

## Methods

### Technology overview: The Community Health Toolkit (CHT)

2wT system was built on a foundation provided by the open-source CHT, a free, open-source digital health global good to advance universal health coverage. The CHT Core Framework, stewarded by Medic and released under AGPL-3.0, is a global good [39] that supports community health workflows. Using the CHT as the basis for 2wT aimed to decrease the time and resources required to build full-featured, reliable, interoperable, secure, and ready-to-scale digital health application (app) rather than code from scratch. Apps built on the CHT Core Framework are highly customizable, support multiple languages, run offline, and work with basic feature phones (via SMS), smartphones, tablets, and computers to support integrated care coordination and delivery. While the CHT is designed for community-level HCW personas, it is highly extensible through integrations with complementary Android apps, electronic medical records, messaging systems, and national health information systems. CHT apps meet various needs, including bidirectional messaging, closed-loop referrals, aggregate reporting and any longitudinal records. In 2022, CHT powered applications supported 22.2 million caring activities and more than 41,216 community-based HCWs in 14 countries across Africa and Asia. The development of 2wT adhered to WHO’s recommended principles for digital development [40].

### Adapting 2wT from VMMC for ART via Human Centered Design

The core components of the target 2wT system were drawn from a previous CHT-based innovation to improve post-operative engagement in care after VMMC [32]. 2wT for VMMC was adapted for the ART context collaboratively by Malawian ART stakeholders. In brief, 2wT for ART intervention included: (1) automated SMS workflows that sent weekly, non-HIV related motivational or educational messages to promote client wellbeing; (2) individually-tailored SMS reminders to clients with upcoming visits, with a response requested; and (3) an open SMS channel for communication between clients and HCWs to allow clients to reschedule visits, report transfers, or get help for other logistics concerns. The 2wT system includes prompts for HCWs to respond to client transfer requests, mute messages, or change visit dates. If clients miss visits, 2wT prompts clients with response-requested reminders. At 14 beyond an expected ART refill visit, the 2wT system creates a prompt to HCWs to refer a client to tracing via Back to Care. All 2wT system data is documented to ease retention reporting. We detailed the 2wT intervention, itself, previously [38].

**Fig 1:**
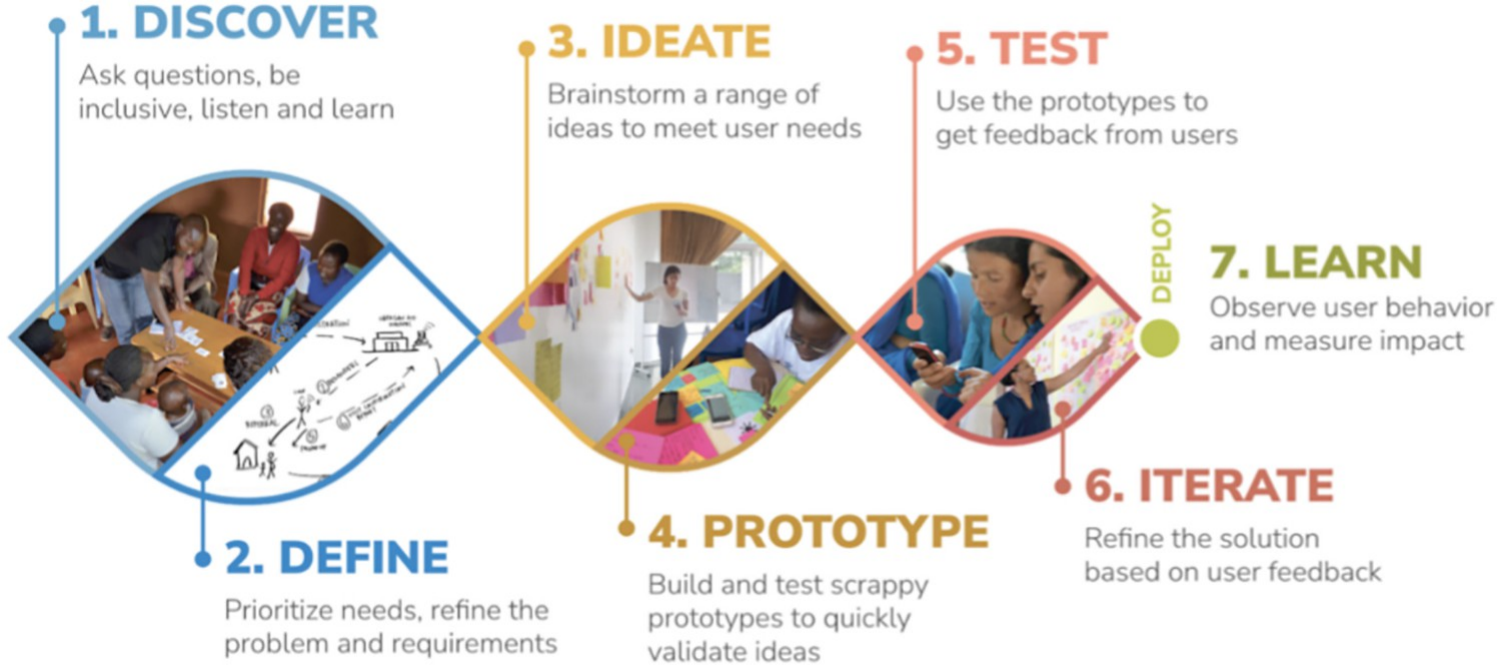
Developing and optimizing 2wT for ART retention: Medic’s human-centered design process [41].

Medic’s continuous HCD approach had seven dynamic, non-linear phases (Fig 1). Medic designers led participatory HCD activities, collaborating closely with a multi-disciplinary team of clients, HCWs, supervisors and software developers to specify the system requirements for the local context. This approach considers the motivations, concerns and behaviour of the end users and process facilitators across the design process, limiting time and resources wasted on creating features that do not benefit, are not relevant, or are unacceptable to the target population [41–46]. At multiple stages, key stakeholders were presented with updated workflow drafts, including features for automatic flags for clients who miss clinic visits, task management and prioritization features, longitudinal client records, data collection forms, routine syncing, and dashboards for routine monitoring. Iterative improvements were informed by analysing users’ system usage patterns and insights gleaned from the feedback sessions.

### Study setting

Lighthouse Trust is a WHO-recognized Centre of Excellence that operates two clinics in urban Lilongwe, Malawi [47]. The 2wT intervention was implemented in the largest Centre of Excellence, MPC, in Bwaila Hospital located in Lilongwe, Malawi. The MPC clinic currently has over 24,000 clients alive in care.

### Participants

The overall 2wT intervention was designed with and for new ART initiates and HCWs at MPC. In this study, HCWs are defined as professional staff involved in diverse healthcare activities, including clinical, retention, monitoring, and counselling activities. HCW participants in the HCD-focused formative research and usability study were selected by purposive sampling to identify participants based on association with retention efforts, including 2wT. For the formative HCD research using informal feedback sessions, eligible HCW participants for this study included key stakeholders; departmental managers; M&E team members (data officers and IT officers); Expert Clients (MPC peer ART mentors); and Back to Care staff, including field tracers and retention officers. For the key informant interviews (KIIs), only HCWs actively engaged in the 2wT implementation were eligible for interviews. Participants across all phases were identified by the retention program coordinator for MPC.

### Data collection

Data collection took place over three distinct phases: 1) informal feedback sessions with 2wT stakeholders, 2) a small pilot, and 3) key informant interviews (KIIs).

### Phase I: formative HCD co-creation research and informal feedback sessions

We conducted four feedback sessions from October to November, 2020. The sessions were undertaken as part of routine program monitoring and evaluation and included various MPC stakeholders involved in client retention (Table 1) to understand their needs and context, informing the 2wT design. In addition to two virtual sessions led by Medic, 2wT HCWs conducted two practice educational sessions with 8 clients (3 men and 5 women) who volunteered to contribute to inform the development and improvement of 2wT recruitment and educational materials. The Medic team compiled and synthesized feedback from the sessions and analysed program reports from 2wT staff.

**Table 1:** Summary table of participants and their roles.

Activity	Participants	Role
Phase I: Formative HCD co-creation research and informal feedback sessions	1. Departmental managers 2. M&E team members (data officers and IT officers) 3. Expert Clients 4. Back to Care staff	1. Oversee the implementation of ART programs at MPC and LH 2. Data users 3. MPC client-to-client ART mentors 4. Coordinate retention in HIV care efforts
Phase II: Evaluation from a small-scale pilot and subsequent 2wT app improvement	1. New ART initiates (within 6 months of starting ART) 2. 2wT HCWs 3. M&E staff 4. Medic teams	1. 2wT system users 2. 2wT system users 3. Data users 4. 2wT system designers and developers
Phase III: Usability perspectives from HCWs post-implementation	Diverse HCWs involves in 2wT implementation or supervision	2wT system users, including 2wT officers, monitoring and evaluation staff, retention team, and supervisors.

### Phase II: Evaluation from a small-scale pilot and subsequent 2wT app improvement

A soft 2wT pilot took place in June 2021 with 50 new ART clients, identifying strengths of the system and key areas of improvement before launch. Results during and after the pilot were discussed with 2wT HCWs, M&E staff and Medic teams via a 5-part series of remote design sessions. The Medic team moderated each one-hour sessions held via Zoom, a web-based video conferencing tool. This series of discussions focused on clients and HCWs feedback and an analysis of system usage trends. Each session built on the previous insights and inputs, focusing on optimizing 2wT for HCWs (system users), M&E and 2wT data users, and clients.

### Phase III: Qualitative exploration of 2wT usability among HCWs, post-implementation

2wT launched in June, 2021. Six months later, we conducted ten KIIs with diverse HCWs from MPC who were involved in 2wT implementation, supervision, M&E, and IT support. KIIs took place over a period of 2 weeks and used an interview guide for consistency. The KII guide was pre-tested and revised by the study team. A trained facilitator from Lighthouse Trust, but external to the 2wT study team, conducted the interviews either at the individual participant’s offices or a place identified as convenient away from other health facility staff, as selected by the interviewee. Participants were recruited, briefed on the purpose of the activity and written consent obtained for recording by those who chose to participate. Each interview session took place in English and lasted for approximately 30 minutes. The KII guide and reports were uploaded to a secured folder only accessible by the study researchers.

### Data analysis

For phases I and II, informal program reports were reviewed and synthesized by the 2wT study team. For the KIIs, we generated verbatim transcripts with Otter.ai software [48]; recordings were deleted once transcribed and the transcripts anonymized. We conducted our analysis using NVivo 12 Pro qualitative text analysis software (QSR International. Burlington, Massachusetts). The research team used a few transcripts to develop and refine a codebook using inductive and deductive approaches and coded all interview scripts. The research team, HCWs (system users), M&E and data users collaboratively reviewed and discussed the resultant codes to formulate themes based on the DEPICT method of participatory qualitative analysis [49].

### Ethics

The parent study, of 2wT client retention outcomes, including the usability and acceptability sub-study components, was reviewed and approved by the University of Washington ethics review board (STUDY000101060) and the Malawi National Health Sciences Research Committee (#20/06/2565). HCW participants in KIIs provided written informed consent. No other identifiers were produced nor utilized for de-identified formative data analysis in phases I or II.

## Results

### Overview: 2wT intervention messaging flow

**Fig 2:**
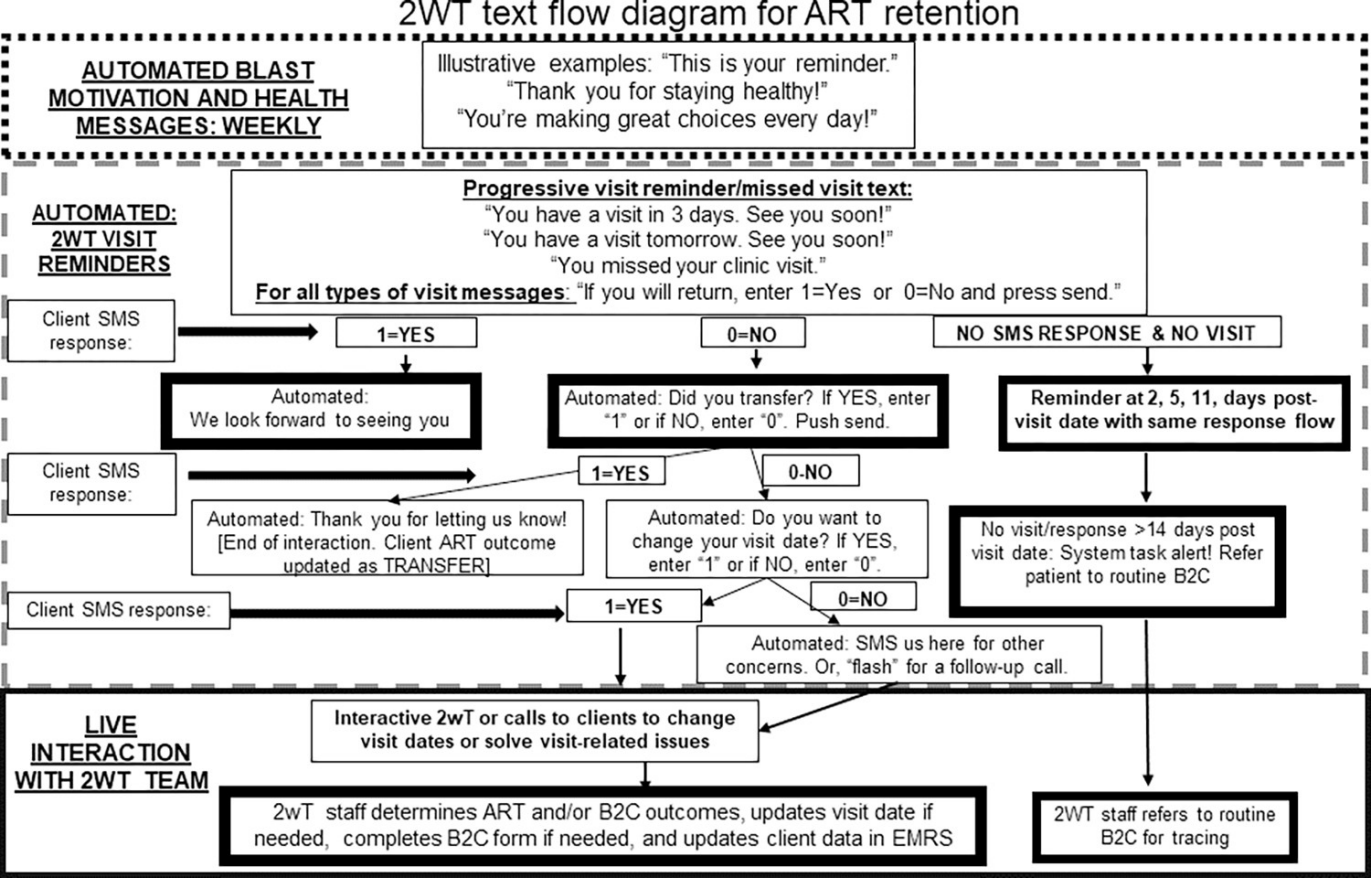
2wT client messaging flow diagram for ART retention [38].

**Fig 3:**
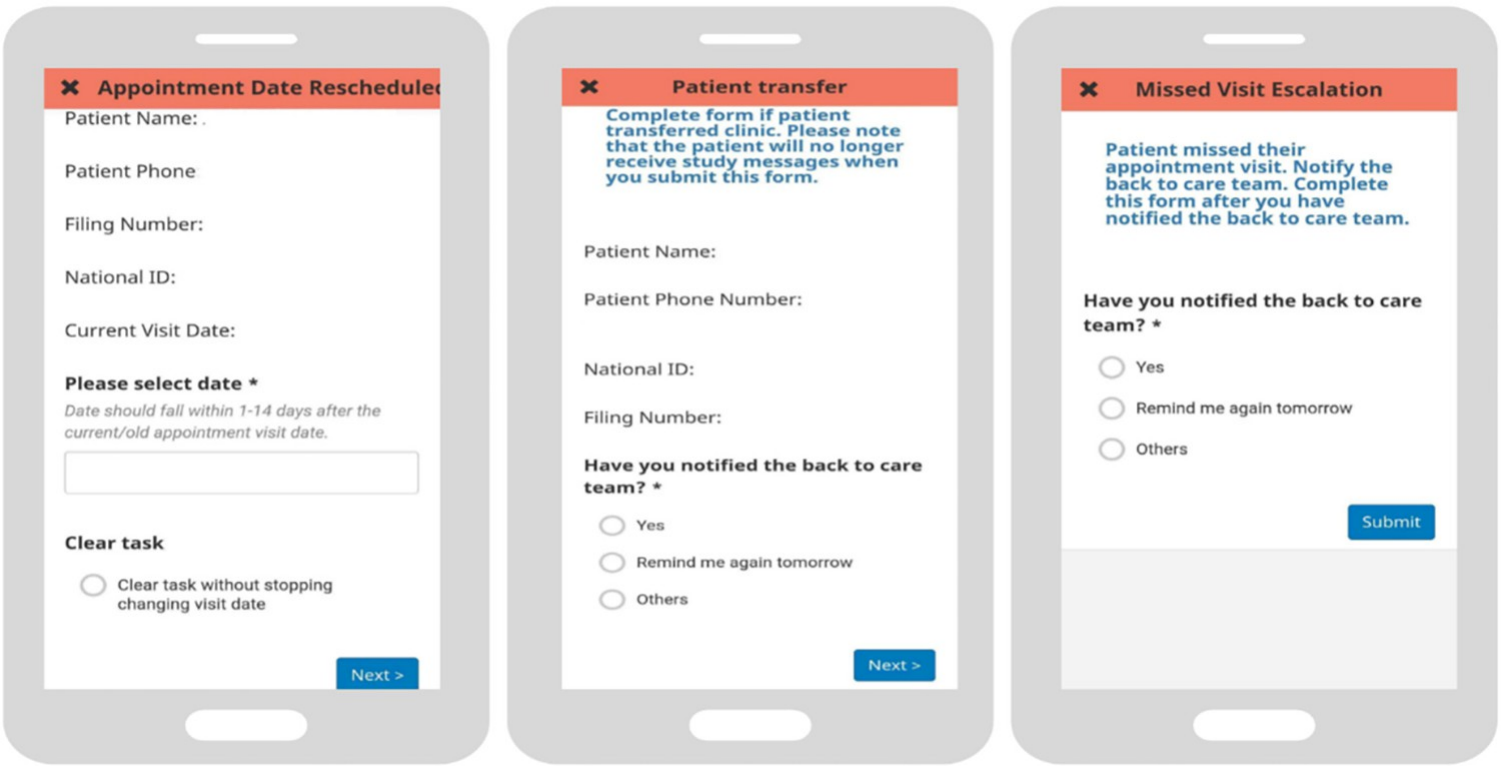
2wT app follow up actions for HCWs.

In brief, the 2wT system (Fig 2) is a hybrid automated and manual texting interaction between clients and HCWs. Weekly motivational messages about general wellness and appointment reminders are sent to all enrolled clients in anticipation of their specific visit with a response requested. Responses to the appointment reminders yield follow up actions for HCWs such as confirming clinic transfers, visit updates and referrals (Fig 3). If a visit is missed, clients receive up to 3 additional reminders until they either return to the clinic or get referred to the Back to Care program for tracing. SMS is free for clients. Messages are sent either in English or Chichewa according to clients’ preferences, but clients can send an SMS at any time in any language. Clients who opt into 2wT can elect to opt out of motivational and/or visit reminder messages at any time. 2wT officers usually respond within 1–2 days. Findings from the client usability and acceptability study were described previously [38].

### Phase I Outcomes: Formative HCD co-creation and informal feedback

Several key areas of client-focused improvements were identified across groups and addressed. First, participation in 2wT for clients was only for those who opted in, reducing risks of confidentiality or disclosure. To improve inclusion of, and acceptability of 2wT for clients, the level of literacy was reduced to a minimum and available in Chichewa and English, as selected by the client. Expert clients helped write, review, revise, and translate adherence and motivational messages; expert clients were identified by HCWs as the best group to conduct 2wT education for new ART clients. 2wT educational materials were augmented to include a client flow chart to guide HCWs in explaining to clients how the message flow worked, how to respond, and how to stop messages. HCWs received a flip book and poster to better explain to clients how to interact with the system with illustrations for participants on how to respond with a “1” or “0” and to improve clarity on SMS communication via free text messages. The neutrality of all text messages was reviewed to ensure that neither reminders nor motivational words would expose the HIV status of the client (e.g., HIV, ART, adherence, clinic, etc.). With the opt-in and clear education for clients before voluntary enrollment, valid concerns were reduced about others including spouses seeing the text messages where there is risk of disclosure or abuse. HCWs also informed the SMS timing for clients who were potential defaulters (those who missed their visits by >14 days), landing on SMS reminders at 3 times after a missed visit and before 14 days. For HCWs, additional sensitization confirmed that 2wT would augment, not replace, other early retention interventions. To reduce redundancy between 2wT-focused interventions and other expert client-led or phone reminder systems, SMS reminders timing would be aligned with other Back to Care program interventions for standardization. Client recruitment would take place during routine enrollment and coincide with routine locator and tracing form completion, reducing impact on MPC’s routine operations. 2wT activity flows and HCW workload were discussed, including discussions of potential workload increases (2wT interaction on the system) and decreases (reduced tracing if clients were maintained in care). As a result, one HCW was designated as the “2wT officer,” and accountable for daily review and responding to client SMS in the morning and afternoon, following up with calls when needed. A set of standard operating procedures for 2wT to EMRS data exchange were created, providing steps to update visit dates, de-identify shared data, increase security, reduce system errors and smooth data flow. Concerns about reminding clients accidentally after they actually attended a visit were diminished due to regular synchronization of data in EMRS and 2WT system to prevent or reduce false referral for missed visit reminders or tracing.

### Phase II Outcomes: Pilot evaluation and subsequent 2wT app improvement

The pilot revealed three main challenges in the EMRS to 2wT data exchange that were addressed via joint working sessions focused on data exchange using automated and manual approaches. One of the challenges was that the resulting process was a one-way flow of data from the EMRS to 2wT allowing only offline EMRS data access for 2wT. The team explored the data pipelines and information needs, including how records would be identified and uniquely mapped to the specific clients files. Protections for client data were strengthened, including data export for consenting participants with limited variables relevant to appointment dates and ART outcomes only. Concerns around dealing with EMRS outages led to the inclusion of paper-based data collection forms which could be used to manually update appointment dates; paper forms were not needed during implementation. The third challenge involved capturing 2wT outcomes retrospectively into the EMRS system. For cost reduction and efficiency, the study team applied for a short code using both common carriers, Airtel and TNM cellular services, for optimization, a decision that allowed all clients to communicate for free via 2wT but delayed the launch of the pilot by several months. Additional concerns about potential delays or failed SMS resulted in several additional M&E processes including measuring the timeliness of SMS interactions, assessment of workload, and message failure rate reviews. System usage analysis of messaging interactions revealed errors in the system such as clients continuing to receive messages after they had transferred clinics and restrictive SMS broadcasting rules that return messages as expired or failed if a phone number is absent on the network or off for over 12 hours.

### Phase III Outcomes: Usability and acceptability perspectives from HCWs post-implementation

#### Participant roles

Of the 10 HCWs who participated in KIIs, 5 were directly involved in the recruitment, education, and/or consenting of 2wT clients who enrolled in the study; several of these HCWs also interacted with clients via the 2wT system. One participant was an IT officer, tasked with support 2wT data quality. Three participants were part of the MPC monitoring and evaluation team, also supporting client retention activities at MPC. One participant was in a supervisory role for members of the 2wT study team.

#### Advantages of the 2wT platform: client-focused benefits

Almost all HCWs noted that the automated client visit reminders helped clients remember their visits, which was highly acceptable to HCWs. KII5 reported that, *“the feedback that we’re getting from participants is that previously they could forget the day they were supposed to come to the clinic*.*”* HCWs also believed that reminder messages encouraged adherence to medications, a useful client support tool, by helping them plan ahead to make time and transport arrangements:

*“They have good reminders*, *they are preparing on time*, *they might be having a problem of transport but because of the reminders it is giving them time to prepare that in the next three days they should look for transport so that they can come to the clinic in time*.*”* [KII5]

HCWs approved of the weekly motivational SMS with non-HIV related health education or uplifting messages, reporting that the feedback they received from clients was positive. “*Apart from being reminded that this is your appointment date there are some other bonuses like they also receive motivational texts*. *So*, *they also learn something in this*. *So*, *they are killing with two or more birds with one stone*.” [KII6] Another put the support expressed by clients succinctly: *“The motivational messages also makes* [make] *them feel that they are not alone*, *that we are together with them in every season of life*.*”* [KII3]

Several HCWs expressed that clients may prefer SMS reminders over phone call reminders as it offers privacy and allows clients to read and respond to messages in their own free time:

*“The back to care team*, *they used to call before the 2wT system*, *maybe three or four times*, *they would call the participant to come for their visit*. *So*, *during those frequent calls to the participants*, *at times*, *they* [patients] *were not at a good place to pick the call*… *with the* [2wT] *intervention it is easier*. *It’s because as I mentioned earlier*, *that even if they call the participants several times*, *but that participant maybe in a group*, *is in a bus*, *cannot respond to those calls*, *but with the intervention*, *the messages*, *they just respond right away*.*”* [KII5]

#### Advantages of 2wT platform: HCW-focused benefits

For HCWs, themselves, there was clear acceptability of the 2wT system as almost all believed that 2wT would reduce their workload in several ways. First, HCWs largely noted the expectation that if 2wT was successful, fewer clients would be referred to Back to Care for missed visits and active tracing:

*“One can already tell the benefit is to say they will reduce our workload in terms of the number of patients that we must chase up to them whether through phone or through field tracing…There are costs that are incurred for us to trace them*, *so that will also probably be reduced*.*”* [KII7]

Also, 2wT provides a list of clients who missed visits by 14 days without having to run a manual query of the EMRS for clients who miss visits. That was perceived as useful improvement for HCWs to reduce daily work. One HCW noted, “*If those patients fail to come*, *these guys*, *the 2wT guys*, *immediately give us* [Back to Care] *those names*. *So*, *we trace those patients before they become defaulters*.*”* [KII8] 2wT efficiency gains could reduce the time needed to begin tracing since Back to Care would be “*notified much faster about a missed visit than waiting to extract the file for them after maybe three weeks or four weeks*” [KII2].

Another KII specified how identifying outcomes proactively via 2wT was easy and could reduce work that would typically be completed by active tracing:

*And we even get some outcomes from them* [2wT clients] *through the SMS interactions (someone had died*, *some are transferred out) from the responses that are given to the 2wT officer*. *So we had some outcomes*, *ART outcomes*, *even before we initiate the tracing*. *We just transferred those outcomes into the tracing forms*.” [KII8]

#### Challenges of 2wT: client-focused

HCWs also considered challenges from the perspective of clients. For example, “*some of them also change* [phone] *numbers*, *which affect the study because it seems as if the patients are not responding to the messages*.*”* [KII4] Although 2wT is opt-in (clients ask to participate), enrollment in the 2wT approach does not guarantee improved visit attendance. Another HCW stated that some clients still may not want to come to the clinic: “*They have read it*. *They have received and read it*, *but they just ignored it*.” [KII7] Another explained the depth of the potential problem:

*“In villages*, *they* [2wT clients] *may keep their phones off for some time*. *I think the challenge would be on the technology side*, *where if we do not have a good percentage of people who do not own their own phones*, *and if they own their own phones*, *and if they keep it on all the times…Therefore*, *that has to be explored*. *Like the behavior in terms of people*, *the cultural behavior of people having their phones on*, *people owning their own phones*, *people changing their phone number*.*”* [KII1]

Several HCWs mentioned that client confidentiality concerns may reduce uptake, noting that some clients worry that messages from the aggregator (short code/sender number) may be recognized or deduced from others, potentially revealing that a client is on ART. One HCW, reflecting on why some clients may not opt into 2wT offered that:

*“Some think that it is not safe in terms of confidentiality*, *especially when they are at home*, *even though we explain to them the way the text messages were designed*. *They still feel like it is not a good thing*. *Maybe there are many people who have access to their phone*? *Some people think a lot and may have questions as to what is going on*.*”* [KII3]

HCWs were disappointed by how many clients were unable to participate in 2wT for failing to meet eligibility criteria including having a phone and basic literacy. This reduced intervention acceptability. One HCW noted their surprise at the number of clients without phones, stating that, “*on the ground*, *with the data* [that we] *have*, *we find that most people they don’t have phones*. *So*, *it means those people that might not benefit from this system*.*”*[KII4] HCWs also expressed that the program’s inclusion criteria such as owning a phone at enrollment and willingness to send and receive text messages excluded clients who would benefit from the intervention, for example, “*some of them do not read or write*. *They are willing but they are failing to be enrolled*.*”* [KII6] Even literate clients may need quality 2wT education at enrollment to help them understand how to interpret, and successfully respond to, the message prompts.

#### Challenges of 2wT: HCW-focused

Although discussion of 2wT strengths intimated widespread acceptability of the intervention, HCWs discussed several key challenges. First, although 2wT may reduce the early retention effort, several HCWs expressed concern that there is potential for increased responsibility and workload to manage the system and interact with clients.

*“My concerns for expanding* [the] *study*, *it will need staff as I said*. *And also workload will be high*. *We will have time to check the texts because we will be receiving a lot of texts on daily basis*. *So they need somebody to be there fully*, *to reply the texts*. *In short it will consume time and the human resource*.*”* [KII6]

Health workers complained about technological challenges, some internal to the system and some due to broader network challenges. Early on, in the first weeks, there were concerns that the system was not sending messages on time, so someone needed to “*check if the messages are able to pass through the systems…the delivery of the messages to the patient*.*”* [KII1] In addition to concerns about the aggregator working correctly, there is the challenge of timely and complete syncing of data from MPC to 2wT data. If the MPC experiences an issue with the EMRS like a power outage that causes visits to not be entered on time or there are delays between EMRS to CHT syncing, “some *participants end up receiving a missed visit escalation*, *a missed visit reminder yet they reported* [attended the clinic visit].*”* [KII2] Many noted that improved integration with the EMRS would help solve several challenges including smoothing exchange between 2wT and EMRS on visit attendance, easing updating of tracing outcomes, and automating updates between systems rather than manual file transfer.

#### Healthcare workers suggestions on 2wT scale-up

Several HCWs noted the need for additional staff if the 2wT intervention expands to include all clients and not new initiates, recognizing that, “*if we are to expand*, *we will be looking at the whole cohort which is too big*, *which means everyone has to pass through this [2wT enrollment] room making it to be a lot of work*.*”*[KII9]

For 2wT system improvements, HCWs suggested that 2wT should enroll multiple numbers per client, including numbers from different telecoms.

*“On the message*, *you’ll find they have used another number*, *on the registration they use another number*, *when they are coming*, *they also use another number*. *Yeah*, *so we need to identify that this is the very same person but how are we going to identify that*? *It’s by asking them when they come for enrollment*. *Do you have other numbers*, *then we can be able to know*.*”* [KII5]

There were also 2wT implementation guidance related suggestions. Several hoped that clients without phones could enroll for 2wT via phones of friends or buddies by allowing clients to enroll in reminders via a trusted number:

*“We would meet some patients who said that “it is my husband who has a phone” or “I live with my mother”*, *so if it was possible that we could be using phones of those that they feel they could disclose to*, *this can make it easy to reach a lot of contacts because those who do not have phones have a guardian with a phone*.*”* [KII10]

To improve 2wT acceptability for HCWs, and to engage more clients who could benefit from 2wT, there will need to be outreach and advocacy to make sure clients know about 2wT. “*I think when it is being scaled up; there will be that sensitization to make people aware of the programme*, *both for the patients*, *and for the providers*, *that they know what is going on*.*”* [KII1] 2wT messages could be further simplified so that very low literacy clients could enroll. One HCW explained:

*“My observation is that not everyone can get involved in* [2wT], *just like another study*, *not everyone is eligible*. *But 2wT is restricted to a certain category of people that have the capability of having a phone…Considering our literacy rate in Malawi*, *and also the economic challenges that we are facing*, *some patients won’t be able to get into 2wT*. *Though they would love to be part of the study*, *but they wouldn’t qualify* [be eligible].” [KII7]

Other interesting comments outside of key themes from HCWs are worthy of consideration. First, although SMS may be less expensive from the program perspective when compared to the time and costs incurred making phone calls or travelling to trace clients in the community, one HCW expressed that what *“has not been really explored more is on the SMS*. *The cost of SMS for Malawi*, *they are quite a bit higher as compared to other countries*.*”* [KII1] Another HCW observed that 2wT might reduce opportunities for in-person counselling since when a client “*missed an appointment*, *he/she is supposed to be counselled by the back to care team*. *Now with texting that means the physical interaction will not be there*, *so no kind of counselling will be provided to the patient for future appointments*.*”* [KII7]

Lastly, HCWs noted that clients may want more information from the 2wT than simply to discuss visits. *“Some patients may have [ART] questions that need to be responded to through the 2wT system*, *but we do not want to discuss clinical things using the system because the personnel* [2wT officer] *in the two-way texting and replying to the texts are not clinical personnel*.*”* [KII9]

## Discussion

Across three distinct phases of formative research activities, we found that putting HCWs at the center of iterative HCD design, adaption, and improvement helped increase 2wT usability and acceptability among HCWs in this routine ART clinic setting in urban Malawi. The process of creating an easy to use 2wT system that met their expressed retention support needs included observing users in their environment, working together to define the problems to be solved, collaborating with users to generate and test ideas, and evaluating the appropriateness of the prototypes with users. These feedback cycles reflects recommendations by the WHO that encourages digital health innovations to reflect local priorities and realities [50], including considerations to reduce burdens on scarce human resources [51] and financial constraints [52]. Likely as a result of inclusion, HCWs believed that 2wT was safe, easy to use, low cost and valuable to them. The use of highly participatory, iterative HCD approaches to assess the 2wT approach throughout the planning, co-creation, prototyping, pilot, and implementation phases also aligns with WHO guidance on digital health monitoring and evaluation [15]. With evidence of 2wT’s positive impact on client retention [53], we share several insights into 2wT strengths, weaknesses, and suggestions for scale that may inform future optimization and expansion of 2wT or similar ART retention apps in the SSA region.

The formative research phases suggest several advantages of the HCD process to make an app both usable and acceptable for HCWs. 2wT software was developed using an existing open-source tool, the Community Health Toolkit (CHT) [54], and leveraged CHT adaptations created for the previous 2wT for VMMC implementation, reducing time for software development. Using the proven CHT framework improved stability and security of the system while previous lessons learned on integration with other apps streamlined the software integration process with the messaging platform, RapidPro [55]. Furthermore, the 2wT design reflected local HCW preferences and their client-focused priorities, likely aiding 2wT buy-in from inception. Similar to other mHealth design feedback, HCWs and stakeholders of the 2wT system appreciated the simplicity of the system for HCWs and their clients, including use of tailored SMS-based messaging (not requiring smartphones) to improve client uptake [56]. Moreover, HCWs liked the hybrid approach that included both automated retention support for clients (blast appointment reminders) and interactive communication between HCWs and clients for individual support. HCWs also felt that they were able to support more clients to remember and plan for their upcoming appointment visits, but with less workload–a critical selling point for the low resource setting.

Investment in the HCD process likely pays longer-term dividends. While HCD processes focus on users’ needs and ensures that feedback is integrated to optimise the app for the local setting, application of the HCD methodology does slow intervention implementation. While short-term delays can be frustrating, HCD minimizes significant product improvements in the long-term and offers the flexibility to make minor iterations during implementation. Multiple design meetings were held with the different stakeholders at the onset of the project to introduce the HCD concept, seek alignment on the HCD methodology and promote buy-in of the HCD process. These steps were necessary to ensure proper mHealth fit to HCW and client needs, aligning with findings from other research emphasizing the importance of engaging stakeholders from the project’s initiation to encourage longer term sustainability potential [57]. Divergent views presented during the co-design sessions were addressed in a series of improvement cycles, building consensus for prioritization of the app features. Consideration of opinions and experiences of Expert Clients, a cadre of supportive HCWs who are living with HIV and on ART at MPC, was also critical to inform app messaging design. Resulting delays for additional message testing translated to improved SMS content and flows for new ART initiates enrolled into 2wT.

Iterative feedback phases also provided HCWs with an opportunity to identify, and overcome, several 2wT challenges that could have reduced usability or acceptability. Recent findings on 2wT effectiveness show improved 15% improved retention among 2wT clients at 12 months [53]; however, HCWs noted that 2wT does require additional work. Monitoring the system, responding to clients via SMS to prevent clients from missing visits, and timely follow-up for those identified by 2wT as potential LTFU requires dedicated personnel time. It is hoped that 2wT cost-effectiveness research, currently underway, would show that 2wT-related workload is offset by economy of scale as 2wT moves from research to expansion. Reduced costs associated with improved retention and preventing tracing of transferred or traveling clients among 2wT clients would free HCW time for other retention support, including 2wT operations. Second, HCWs were concerned about client eligibility and access as 2wT required ownership or primary use of a mobile phone and enough literacy to use SMS. Previous research in Malawi suggests that simply owning a phone does not necessarily equate with digital literacy to use the device for healthcare-related purposes [58]. Echoing findings from a recent review of client perspectives on mobile health [59], HCWs feedback in this setting suggested further reduction of message complexity to cater for clients with low literacy, allowing of shared phones to promote access, and enrolling all interested clients (not just new initiates) for future expansion. HCWs also noted that enhanced 2wT client education would help confirm that clients with low literacy levels understand the system prompts and are empowered to reach out to HCWs when need arises. Lastly, lack of 2wT integration with EMRS was highlighted as a limitation to successful expansion. Integration with existing electronic health (eHealth) infrastructure is recommended by WHO’s Global strategy on digital health 2020–2025 [60]. But successful integration depends on multiple elements outside the control of the Lighthouse Trust and its 2wT intervention environment, including an enabling environment for integration of digital health into a nation’s healthcare system; comprehensive eHealth policy; and sufficient capacity for eHealth interoperability management.

Several suggestions could help bring 2wT more smoothly to scale. 2wT is intended to complement, and not replace, other ongoing retention activities. Once established as effective, 2wT should be included as part of comprehensive retention efforts, considered for linkage and retention in ART care, allowing clients to choose a retention support system (2wT, phone calls or expert client home visits) that meets their needs. Not all clients restrict their SMS to visit related information; developing a triage and referral for clinical questions from 2wT clients to the Lighthouse Trust call center is worthy of consideration. Technological considerations such as the use of symbols or emojis could address some of the limitations related to literacy thereby improving user engagement. On the technology side, dashboard development should streamline client tracking while completion of the interoperability pipeline between 2wT and the EMRS would smooth daily updates to client visits and ART status. Future consideration of artificial intelligence or natural language processing could also support imputation of next visit dates, creation and curation of the motivational messages, reduce workload, and efficiently triage for follow-up.

## Limitations

The formative study focussed primarily on HCW experiences and not clients. Findings from the client usability testing were described previously [38]. Some of the challenges experienced while implementing HCD for this project were influenced by COVID19 travel restrictions, including the shift from in-person field visits to remote user engagement sessions, contributing to delays that could have influenced HCW perspectives. Medic led the design and development of the 2wT system and was part of the research team. Formative work in intervention development is inherently biased towards creating momentum for continued iterations and improvements; however, the authors and analysts worked to create balance in reporting both success and weaknesses of the 2wT approach. Despite these limitations, emphasis on the role of formative research and centering of HCWs in HCD is an important contribution to continued strengthening of locally-relevant and -optimized digital health innovations.

## Conclusion

HCWs have specific needs and valuable visions of how digital health tools could aid in easing their workload, demonstrating the value of HCW inputs into the HCD process. Engaging HCWs throughout the co-design and co-creation process promotes ownership and acceptability, informing software that responds to the specific needs and challenges of the contexts. The iterative HCD process aims to combat an all-too-common digital health problem of piloting, but not problem solving, to overcome weaknesses and build on strengths for maintained mHealth use. The cyclical adaptation of the 2wT platform for direct HCW to client engagement moved from a short term intervention (voluntary medical male circumcision, 14 days follow-up) to a longer term intervention (ART care, lifelong follow-up), providing an opportunity to consolidate lessons learned and build a stronger, more flexible 2wT system that could strengthen direct communication pathways for longitudinal care. The success of 2wT for improved retention could lead to adaptation for other health care contexts that could benefit from similar, appropriate mHealth interventions for routine, low-resource settings.

## Supporting information

S1 Table Emergent qualitative themes from HCW interviews. (TIF)
