# Supplementary figures and images for: Centering healthcare workers in digital health design: Usability and acceptability of two-way texting to improve retention in antiretroviral therapy in a public HIV clinic in Lilongwe, Malawi
Source: PLOS Digit Health. 2024 Apr 3;3(4):e0000480. doi: 10.1371/journal.pdig.0000480 (PMC10990210; doi:10.1371/journal.pdig.0000480)

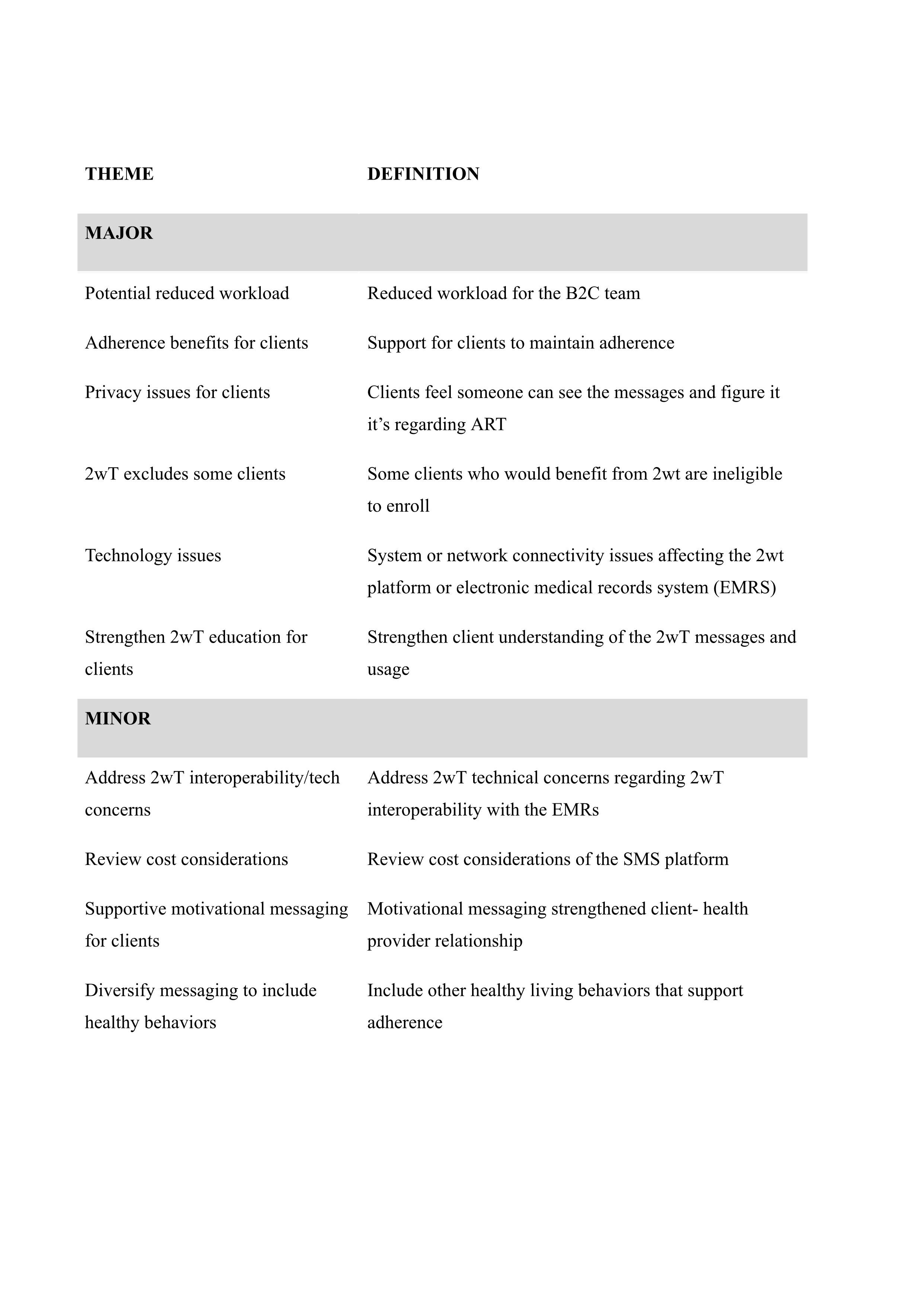

Supplement: S1 Table — (TIF) [file pdig.0000480.s001.tif]
